# Prehospital antimicrobial use among people with coronavirus disease 2019 (COVID-19) in Uganda

**DOI:** 10.1017/ash.2022.238

**Published:** 2022-06-10

**Authors:** Felix Bongomin, Andrew Marvin Kanyike, Phillip Musoke, Senai Goitom Sereke, Jerom Okot, Winnie Kibone, Andrew Weil Semulimi, Joseph Baruch Baluku, Frederick Nelson Nakwagala

**Affiliations:** 1 Department of Medical Microbiology, Faculty of Medicine, Gulu University, Gulu, Uganda; 2 School of Medicine, College of Health Sciences, Makerere University, Kampala, Uganda; 3 Department of Internal Medicine, Faculty of Health Sciences, Busitema University, Mbale, Uganda; 4 Makerere Lung Institute, College of Health Sciences, Makerere University, Kampala, Uganda; 5 Division of Pulmonology, Kiruddu National Referral Hospital, Kampala, Uganda; 6 Department of Internal Medicine, Mulago National Referral Hospital, Kampala, Uganda

*To the Editor*—Empirical use of antimicrobial agents has been in routine practice since the onset of the coronavirus disease-2019 (COVID-19) pandemic.^1^ Even though COVID-19 is caused by a virus, health care practitioners have been equivocally prescribing different antibiotics to suspected and confirmed COVID-19 patients^2^ because of the presence of comorbid conditions and suspicion of superimposed community-acquired pneumonia.^3,4^ However, the global prevalence of bacterial superinfection in COVID-19 patients is much lower than the practice of empiric use of antimicrobials (1%–16% vs 72%) among people with COVID-1.^4,5^

Different antimicrobial agents, antivirals, antimalarial agents (eg, hydroxychloroquine) and antiparasitic agents (eg, ivermectin) have been used globally to treat people with COVID-19.^4–6^ This superfluous use of antimicrobials is not given much attention, yet it can lead to increasing incidence of antimicrobial resistance that is already a global threat. Despite widespread use of azithromycin and other antimicrobials among people hospitalized with COVID-19 in Uganda, the burden of prehospital antimicrobial use among this population is unknown.

We carried out a cross-sectional study using interviewer administered semistructured questionnaires to determine the prevalence of prehospital antimicrobial use and associated factors among persons hospitalized with COVID-19 at 2 large COVID-19 treatment units (CTU)—Mulago National Referral Hospital and Namboole Stadium—in Uganda, with bed capacities of >3,000 beds, catering to a range of patients from all over the country, between July and August 2021. The data were analyzed using STATA version 16 software (StataCorp, College Station, TX), and the study was approved by Mulago Hospital Research and Ethics Committee (approval no. MHREC 2097).

In total, 110 participants were enrolled, with median age of 37 years (IQR, 28–51). Among them, 62 (56.4%) were female, 59 (53.6%) Catholic, 80 (72.7%) were from the central region of the country, 68 (61.8%) were formerly employed at Namboole Stadium CTU and 64 (58.2%) were employed at Namboole Stadium CTU. Among the participants, 61 (55.4%) were unvaccinated for COVID-19. Of the 49 (44.6%) who had been vaccinated, 29 (60.4%) had received only 1 dose. Prior to admission, 44 participants (40%) were reported to be on treatment for COVID-19. Prehospital antimicrobial use was reported by 96 patients (87.3%), and 91 (94.8%) of these were antibiotics.

Azithromycin alone (n = 41) or in combination with other antibiotics (n = 8) was the most used antibiotic (52.1%). Moreover, 27 participants (28.7%) were on either intravenous ceftriaxone alone (n = 15) or in combination with other antibiotics (n = 11), or on piperacillin-tazobactam (n = 1). At bivariate analysis, preadmission treatment for COVID-19 (*P* = .007), religion (*P* = .008), and the CTU site (*P* < .0001) were related to prehospital antimicrobial use (Table 1). On multivariate logistic regression participants who initiated treatment before admission at a CTU had 10-fold higher odds of prehospital antimicrobial use than those who started treatment at the CTU (adjusted odds ratio, 10; 95% confidence interval [CI], 1.3–26.7; *P* = .026).

**Table 1.** Bivariate Analysis for Sociodemographic Factors Associated With Prehospital Antimicrobial Use

Variable	All (n=110), No. (%)	Prehospital Antimicrobial Use		*P* Value
		No (n=14), No. (%)	Yes (n=96), No. (%)	
Age, median y, (IQR)	37 (28–51)	36.5 (28–46.3)	37 (29–52)	.704
<38	55 (50)	7 (50)	48(50)	1.000
≥38	55 (50)	7 (50)	48(50)	
**Sex**				
Female	62 (56.4)	8 (57.1)	54(56.3)	1.000
Male	48 43.6)	6 (42.9)	42(43.8)	
**Region of residence**				
North	3 (2.7)	0 (0)	3 (3.1)	.725
Western	7 (6.4)	1 (7.1)	6 (6.3)	
Central	80 (72.7)	12 (85.8)	68 (70.8)	
East	20 (18.2)	1 (7.1)	19 (19.8)	
**Level of education (n=108)**				
Primary	19 (17.6)	2 (14.3)	17 (18.1)	.430
Secondary	33 (30.6)	2 (14.3)	31 (33)	
Tertiary	40 (37.0)	7 (50)	33 (35.1)	
None	16 (14.8)	3 (21.4)	13 (13.8)	
**Formal employment status**				
Employed	68 (61.8)	9 (64.3)	59 (61.5)	1.000
Not employed	42 (38.1)	5 (35.7)	37 (38.5)	
**Religion**				
Anglican	24 (21.8)	3 (21.4)	21 (21.9)	**.008**
Born again	7 (6.4)	0 (0)	7 (7.3)	
Catholic	59 (53.6)	7 (50)	52 (54.1)	
Islam	17 (15.5)	1 (7.1)	16 (16.7)	
SDA	3 (2.7)	3 (21.4)	0 (0)	
**Marital status**				
Married	58 (52.7)	8(57.1)	50(52.1)	1.000
Divorced	10 (9.1)	1(7.1)	9(9.4)	
Single (never married)	33 (30)	4(28.6)	29(30.2)	
Widowed	9 (8.2)	1(7.1)	8(8.3)	
**Treatment site**				
Mulago CTU	46(41.8)	0 (0)	46 (47.9)	**<.0001**
Namboole CTU	64(58.2)	14 (100)	50 (52.1)	
**Preadmission treatment for COVID-19**				
Yes	66(60)	13 (92.9)	53 (55.2)	**.007**
No	44(40)	1 (7.1)	43 (44.8)	
**Concurrent herbal medical use (n=100)**				
No	73 (73)	3 (74)	70 (72.9)	1.000
Yes	27 (27)	1 (25)	26 (27.1)	
**COVID-19 vaccinated status**				
Not vaccinated	61 (55.5)	10 (71.4)	51 (53.1)	.256
Vaccinated	49 (44.5)	4 (28.6)	45 (46.9)	
**Doses received (n=48)**				
1	29 (60.4)	4 (100)	25 (56.8)	.142
2	19 (39.6)	0 (0)	19 (43.2)	

Note. IQR, interquartile range. Significant *P* values are shown in bold.

This study revealed a high percentage (87.3%) of prehospital antimicrobial use, with almost half of the antimicrobials prescribed by healthcare practitioners. These findings are similar to those of studies conducted during the first wave of the pandemic in Wuhan, China,^7^ and Bangladesh.^4^ Although nosocomial bacterial and fungal infections may develop in during the course of COVID-19 among hospitalized patients, superinfections with these pathogens are very uncommon among patients in the outpatient settings.^8^ To mitigate the overprescription of antimicrobials, the World Health Organization has made available a new tool—AWaRe (Access, Watch and Reserve).^9^ The high prevalence of prehospital use among Ugandans diagnosed with COVID-19 highlight the need of heightened awareness on the AWaRe tool and the general aspect of antimicrobial stewardship among the general population and healthcare workers alike. Antimicrobial use for prophylaxis in mild cases of COVID-19 is strongly discouraged by the WHO guideline on clinical management of COVID-19.

However, in moderate-to-critical cases of COVID-19 with clinically suspected bacterial or fungal coinfection, the guidelines encourage the use of antimicrobials.^10^ In our study, most patients took oral azithromycin, which is consistent with study done in Bangladesh,^4^ followed by intravenous ceftriaxone but no antivirals were used. Antimicrobial stewardship program that promote and oversee the safe and judicious use of antimicrobials should be implemented to safeguard the antimicrobials use for feature use and decrease the advent of antimicrobial resistance. Moreover, healthcare workers should be familiar with the WHO AWaRe tool to curtail the increasing concern of postpandemic antimicrobial-resistance–related morbidity and mortality.

In conclusion, prehospital antimicrobial use, including intravenous antibiotics is common among patients with COVID-19, particularly among those who initiated COVID-19 treatment while at home. This antimicrobial overuse can have a devastating consequence in terms of accelerated antimicrobial resistance.
